# Origins and evolution of CRISPR-Cas systems

**DOI:** 10.1098/rstb.2018.0087

**Published:** 2019-03-25

**Authors:** Eugene V. Koonin, Kira S. Makarova

**Affiliations:** National Center for Biotechnology Information, National Library of Medicine, Bethesda, MD 20894, USA

**Keywords:** adaptive immunity, mobile genetic elements, signalling, gene shuffling

## Abstract

CRISPR-Cas, the bacterial and archaeal adaptive immunity systems, encompass a complex machinery that integrates fragments of foreign nucleic acids, mostly from mobile genetic elements (MGE), into CRISPR arrays embedded in microbial genomes. Transcripts of the inserted segments (spacers) are employed by CRISPR-Cas systems as guide (g)RNAs for recognition and inactivation of the cognate targets. The CRISPR-Cas systems consist of distinct adaptation and effector modules whose evolutionary trajectories appear to be at least partially independent. Comparative genome analysis reveals the origin of the adaptation module from casposons, a distinct type of transposons, which employ a homologue of Cas1 protein, the integrase responsible for the spacer incorporation into CRISPR arrays, as the transposase. The origin of the effector module(s) is far less clear. The CRISPR-Cas systems are partitioned into two classes, class 1 with multisubunit effectors, and class 2 in which the effector consists of a single, large protein. The class 2 effectors originate from nucleases encoded by different MGE, whereas the origin of the class 1 effector complexes remains murky. However, the recent discovery of a signalling pathway built into the type III systems of class 1 might offer a clue, suggesting that type III effector modules could have evolved from a signal transduction system involved in stress-induced programmed cell death. The subsequent evolution of the class 1 effector complexes through serial gene duplication and displacement, primarily of genes for proteins containing RNA recognition motif domains, can be hypothetically reconstructed. In addition to the multiple contributions of MGE to the evolution of CRISPR-Cas, the reverse flow of information is notable, namely, recruitment of minimalist variants of CRISPR-Cas systems by MGE for functions that remain to be elucidated. Here, we attempt a synthesis of the diverse threads that shed light on CRISPR-Cas origins and evolution.

This article is part of a discussion meeting issue ‘The ecology and evolution of prokaryotic CRISPR-Cas adaptive immune systems’.

## 1. Introduction

Thanks to the unprecedented success of the Cas9, Cas12 and Cas13 endonucleases as genome editing tools, during the last decade, biochemical activities, structures, comparative genomic and at least some of the biological functions of CRISPR (Clustered Regularly Interspaced Short Palindromic Repeats)-Cas (CRISPR-associated proteins) systems and individual Cas proteins have been studied in exquisite detail [1–10]. The CRISPR-Cas are adaptive (acquired) immune systems that store the memory of encounters with foreign DNA, primarily that of mobile genetic elements (MGE), in unique spacer sequences derived from MGE and inserted into CRISPR arrays. The transcripts of the CRISPR spacers are used to recognize the cognate sequences and direct Cas nucleases to their unique target sites upon new encounters with familiar MGEs, resulting in the inactivation of the latter.

Like all defence mechanisms, CRISPR-Cas systems evolve in the regime of a perennial arms race with MGE, which results in the rapid evolution of some of the *cas* gene sequences, primarily effector module components [11], and remarkable diversification of the gene composition and organization of the CRISPR-*cas* loci. This molecular diversity underlies the diversification of the molecular mechanisms of CRISPR-mediated defence [8,12,13].

Along with eukaryotic RNA interference (RNAi) and prokaryotic Argonaute-centred defence mechanisms, the CRISPR-Cas belong to nucleic acid-guided defence systems [14–18]. Arguably, among these mechanisms, CRISPR-Cas systems are the most biologically complex because, in contrast with the innate immunity mechanisms, such as those of the Argonaute-based systems and most of the forms of eukaryotic RNAi, but similarly to the piRNA branch of RNAi, CRISPR-Cas possess an integral capacity of creating immune memory and thus represent bona fide adaptive immunity [19–22].

Complete CRISPR-*cas* loci consist of a CRISPR array, that is, two to several hundred direct, often partially palindromic, normally exact repeats (25–35 bp each), separated by unique spacers (typically 30–40 bp each), and the adjacent cluster of multiple *cas* genes which are organized in one or more operons encoding both the adaptation and the effector modules, often along with accessory genes [13,23]. The CRISPR-Cas immune response includes three distinct but often intertwined stages: (i) adaptation, (ii) pre-crRNA (pre-CRISPR RNA) expression and processing, and (iii) interference.

During the adaptation stage, a complex of Cas proteins binds to a target DNA molecule and, typically, after encountering a distinct, short (2–4 bp) motif known as PAM (Protospacer-Adjacent Motif), introduces two double-strand (ds) breaks into the target DNA. The released segment, the protospacer, is then inserted between two repeats in the CRISPR array (most often, into the proximal repeat unit that immediately follows the leader sequence), so that it becomes a spacer [24,25]. The CRISPR array is then repaired by the cellular repair machinery, resulting in the duplication of the proximal repeat [26–28]. Some CRISPR-Cas systems employ an alternative mechanism of adaptation, namely, spacer acquisition from RNA (transcripts of a DNA genome of an MGE) via reverse transcription by a reverse transcriptase (RT) that is encoded in the CRISPR-*cas* locus and, in most cases, fused to the Cas1 protein [29,30].

At the expression-processing stage, the CRISPR array is typically transcribed into a single, long transcript, the pre-crRNA, that is processed to generate mature crRNAs by a distinct complex of Cas proteins, a dedicated processing nuclease (Cas6), a single large Cas protein or an external RNase [31,32].

At the final, interference stage, the crRNA that remains bound to the processing complex is employed as the guide (gRNA) to recognize the protospacer or a closely similar sequence in the genome of a virus or a plasmid which is then cleaved and inactivated by a Cas nuclease which can be either a component of the same effector complex or a separate Cas protein [33,34].

The brief description above is an over-simplified scheme that, out of necessity, misses many important details of CRISPR-Cas functions. Such details can be found in numerous recent reviews on different facets of CRISPR-Cas biology [1–9,24,25,31–34].

At both the structural and the functional levels, the CRISPR-Cas systems have a distinct modular organization [13,23]. The two principal components of the CRISPR-Cas systems are the adaptation and effector modules. In most of the CRISPR-Cas systems, the adaptation module consists of the Cas1 and Cas2 proteins, which form a complex in which Cas1 is the enzymatically active subunit, namely, the endonuclease (integrase) involved in the cleavage of both the source, protospacer-containing DNA and the CRISPR array, whereas Cas2 forms the structural scaffold of the complex [24,35–37]. In many CRISPR-Cas systems (see below), additional Cas proteins, such as Cas4, Cas3, Cas9 or RT, also contribute to the adaptation stage, in some cases forming fusions with Cas1 or Cas2 [30,38–40]. In contrast with the comparatively simple and uniform architecture of the adaptation module, the effector modules are highly diverse among CRISPR-Cas systems, and their variation forms the basis of the current CRISPR-Cas classification [8,13], which is described in the next section.

The extraordinary, compared with other defence systems in prokaryotes, complexity and diversity of CRISPR-Cas systems implies a complex evolutionary history. Phylogenomic studies have revealed a pervasive trend in CRISPR-Cas evolution, namely, the contributions of several classes of MGE to the ultimate origin and the subsequent diversification of the CRISPR-Cas systems, in particular the adaptation modules [4,13,23,41–43]. However, the origin of the most prevalent forms of the effector modules remains a much harder problem. In this article, we attempt to synthesize the available clues on the origins and evolution of different components of CRISPR-Cas systems along with the evidence of the reverse trend, that is, recruitment of CRISPR-Cas and their components by MGE.

## 2. Diversity, classification and evolutionary modularity of CRISPR-Cas systems

**Figure 1.**
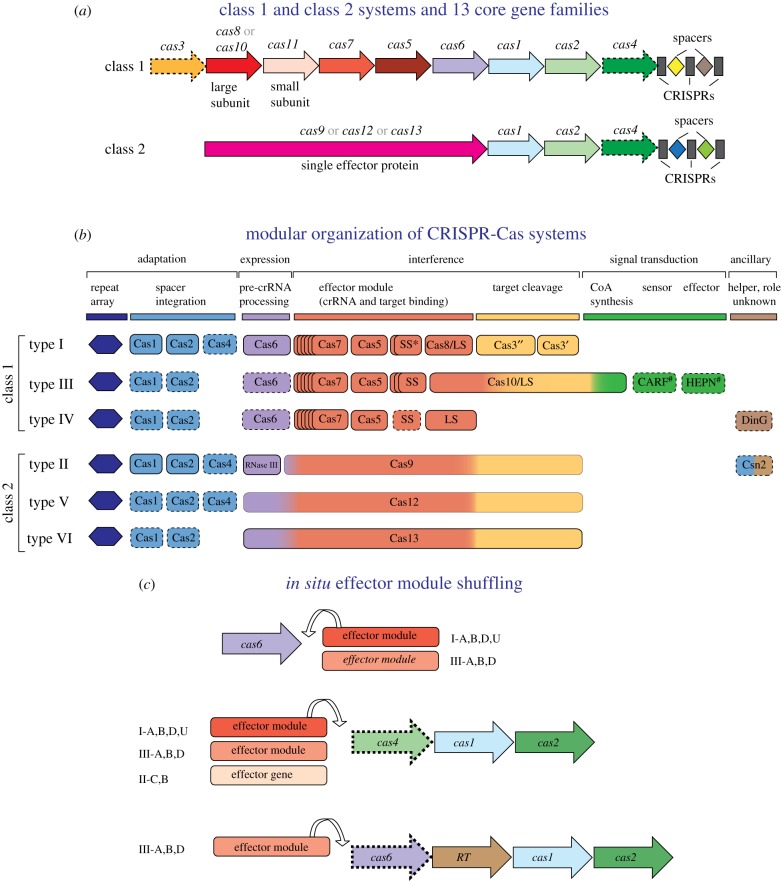
Class 1 and class 2 CRISPR-Cas systems: key features, modular organization and module shuffling. (*a*) The general architectures of class 1 (multiprotein effector complexes) and class 2 (single-protein effector complexes) CRISPR-Cas systems. Genes are shown as arrows; homologous genes are shown by the same colour. Gene names follow the current nomenclature and classification [8,13]. (*b*) The principal building blocks of CRISPR-Cas system types. An asterisk indicates the putative small subunit (SS) that might be fused to the large subunit in several type I subtypes [13]. The # next to the CARF and HEPN domain labels indicates that other unknown sensor and effector domains can be involved in the signalling pathway. Dispensable genes are indicated by a dashed outline. The bottom panel schematically shows module shuffling in CRISPR-*cas* loci. (Online version in colour.)

The CRISPR-Cas systems are a universal immune mechanism that, at least in principle, can adapt to defend the host from any MGE. Because of this universal adaptability, CRISPR-Cas systems do diversify as extensively as innate immune systems, such as restriction-modification modules, the ubiquitous and most abundant defence component in archaea and bacteria. Nevertheless, the Cas protein sequences and the genomic organization of CRISPR-*cas* loci display substantial diversity. All CRISPR-Cas systems are divided into two distinct classes, on the basis of the design principles of the effector modules. Class 1 systems have multisubunit effector complexes comprising several Cas proteins, whereas in class 2 systems, the effector is a single, large, multidomain protein [13] (figure 1). Classification of CRISPR-Cas systems is a complicated matter. There are no universal Cas proteins that could be used as phylogenetic markers, and even the phylogeny of the most evolutionarily conserved protein, Cas1, fails to adequately represent the relationships between CRISPR-Cas systems owing to the semi-independent evolution of different modules (see below). Therefore, the existing classification of CRISPR-Cas systems employs multiple criteria including signature *cas* genes, organization of the *cas* operons and phylogenies of conserved Cas proteins.

The two CRISPR-Cas classes are divided into three types each, types I, III and IV in class 1, and types II, V and VI in class 2; each type is characterized by distinct architectures of the effector modules that include unique signature proteins (figure 1). Each type is further classified into multiple subtypes that are distinguished by subtler differences in locus organization and, also, often encode subtype-specific Cas proteins [8,12,13,43]. The mechanisms of pre-crRNA processing in class 1 and class 2 CRISPR-Cas systems notably differ. In class 1 systems, the maturation of crRNAs is catalysed by a dedicated complex of multiple Cas proteins that was first identified in subtype I-E and designated Cascade (CRISPR-associated complex for antiviral defence) [44–48]. The Cascade complex binds the pre-crRNA and recruits an additional Cas protein, Cas6 (or, on rarer occasions, Cas5), which is the nuclease directly responsible for processing. In type II systems, the prototype of class 2, processing is catalysed by an external bacterial enzyme, RNAse III, with the help of an additional RNA species, the *trans*-acting CRISPR (tracr)RNA, encoded within the CRISPR-*cas* locus [49–54]; tracrRNAs have been identified also in subtype V-B systems, although in this case, the cleavage enzyme remains uncharacterized [53–55]. In types V and VI, pre-crRNA processing is catalysed by a distinct, still incompletely characterized nuclease activity of the same large effector protein that is involved in target cleavage [56–59].

Major differences between class 1 and class 2 CRISPR-Cas systems are apparent also at the interference stage. In type I systems, the processing complex containing the mature crRNA recognizes the protospacer sequence in the target and recruits an additional Cas protein, Cas3, which consists of a helicase domain that unwinds the target dsDNA and the nuclease domain directly responsible for the cleavage [60–62]. In type III, the nuclease involved in the target cleavage is a subunit of the processing complex itself; in this case, no helicase is involved but DNA cleavage requires primary cleavage of RNA transcripts of the target genome by a distinct CRISPR-associated RNase. In class 2 systems, cleavage is performed by the nuclease domain(s) of the large effector protein [49,50,52,54,59,63–69] (see more below).

The adaptation and effector modules of the CRISPR-Cas systems show pronounced autonomy not only with respect to functions and structure, but also evolutionarily (figure 1). The topology of the phylogenetic tree of Cas1, the key subunit of all adaptation complexes and repeat structures, are poorly compatible with the overall classification of the effector modules and phylogenies of individual effector proteins, apparently because of frequent module exchange among CRISPR-Cas systems of different types and subtypes [13]. Numerous examples of such exchange have been reported [30,70–72], including those that occur *in situ*, in the vicinity of several ‘attractor’ genes, namely, *cas6* and various combinations of the adaptation genes *cas1*, *cas2*, *cas4* and *RT* (figure 1). In most cases, the adaptation module, together with the cognate CRISPR array and/or *cas6* gene, remains fixed, as judged by the conservation in related genomes, whereas the effector module genes are shuffled [72]. This *in situ* exchange often involves not only effector modules from different variants of the same CRISPR-Cas subtype but also other subtypes or even other types (figure 1). Type III loci are especially prone to such module recombination: in particular, RT-containing type III adaptation modules have been shown to almost freely combine with effector modules from diverse subtypes of type III systems [30]. The evolutionary pressure to exchange effector modules is likely a consequence of an arms race against rapidly evolving viruses. Viruses encode many anti-CRISPR proteins (Acrs) which so far have been shown to target only components of effector complexes and, typically, show high specificity towards particular variants of CRISPR-Cas systems [73–80]. Exchange of effector modules could provide an escape route from Acrs. The partial evolutionary independence of the adaptation and effector modules is further corroborated by the presence, in numerous bacterial and archaeal genomes, of stand-alone adaptation and, even more often, effector modules [13,43,72]. Notwithstanding all these manifestations of the modularity of the CRISPR-Cas systems, it should be noted that the functional separation of the modules is but an approximation because some Cas proteins, in particular class 2 effectors, appear to be involved in all three stages of the CRISPR response [81,82].

## 3. Origin of the adaptation module and adaptive immunity in prokaryotes from a distinct group of transposons

Genomic surveys of *cas* genes show that Cas1, the endonuclease responsible for spacer integration into CRISPR arrays, is not always encoded within CRISPR-*cas* loci [23]. Examination of the genomic neighbourhoods of ‘stand-alone’ *cas1* homologues has led to the unexpected finding that these *cas1* genes actually are embedded within 12–18 kb regions of genomic DNA flanked by terminal inverted repeats (TIRs) and thus clearly resembling transposable elements [83]. These predicted transposons share two universal genes encoding, respectively, Cas1 and a family B DNA polymerase, and also encompass variable sets of additional genes, mostly encoding diverse nucleases and DNA-binding proteins containing helix–turn–helix (HTH) domains. The biochemical mechanisms of the reactions catalysed by Cas1 during spacer integration into CRISPR arrays and by integrases during transposon integration are closely similar, which naturally leads to the prediction that Cas1 is the integrase of the newly identified group of transposons; accordingly, these predicted transposons were named casposons and their predicted integrases were dubbed casposases [83,84]. Given the invariable presence of a DNA polymerase, casposons appear to be self-synthesizing transposons that direct their own replication during transposition via a copy-and-paste mechanism. This type of transposon so far has not been found in prokaryotes but is common among eukaryotes, many of which harbour polintons which also encode a B family DNA polymerase along with a retrovirus-type integrase that is unrelated to Cas1 [85–87]. The integrase activity of the casposase was promptly confirmed experimentally [88], and moreover, it has been shown that casposons and CRISPR spacers insert into similar target sites [89].

Although casposons are not among the most abundant classes of MGE in prokaryotes, comparative analysis of their gene organization revealed considerable diversity and resulted in the identification of four distinct casposon families which are integrated mostly into archaeal genomes as well as those of some bacteria [90]. Notably, a small group of casposons encode a predicted virus capsid protein, indicating that some of these elements are actually ‘caspoviruses’, in a close analogy to the polintons which also possess capsid proteins and are predicted to form virions [91]. Transposition of casposons has not yet been demonstrated directly but comparative genomic analysis of many strains of the archaeon *Methanosarcina mazei* has led to the identification of clear signs of recent mobility, indicating that at least some of the casposons are active transposons [92].

The phylogenetic tree of the Cas1 family splits into two major branches, one of which includes the casposases and the other one consists of the CRISPR-associated Cas1 proteins [83]. Although, technically, the root position is unknown, this tree topology is compatible with a founding role of the casposase in the CRISPR-Cas evolution. The entire CRISPR adaptation module likely originated from a casposon which could have also contributed additional *cas* genes [41]. Although currently known casposons do not encode Cas2 (the key structural subunit of CRISPR adaptation complexes), some encode nucleases homologous to Cas4, a component of the adaptation module in several CRISPR-Cas subtypes, as well as additional nucleases [83,84]. The ancestral casposon configuration including a gene for a Cas2 homologue can be expected to appear in the growing microbial genome datasets. Additionally, the ancestral CRISPR repeats and the leader sequence could evolve from either the TIRs or a duplicated target site of the ancestral casposon [90].

The event that gave rise to the adaptation module and, concomitantly, prokaryotic adaptive immunity could have involved a chance insertion of a casposon into the vicinity of an ancestral innate immunity locus, followed by immobilization of the casposon and elimination of some of its genes, including the DNA polymerase [41]. The ancestral innate immunity system that gave rise to the CRISPR effector module might have functioned by directly engaging guide RNA derived from transcripts of foreign genomes, in an analogy to prokaryotic Argonaute-centred defence systems [16,18]. However, innate immunity systems homologous to CRISPR-Cas effector modules have not been so far identified in bacterial or archaeal genomes, and thus the ancestry of the effector module remains a hard puzzle [23,53]. In the next section, we consider some clues that might allow us to glean a solution.

## 4. Ancestral class 1 effector modules: origin from a stress-response system?

**Figure 2.**
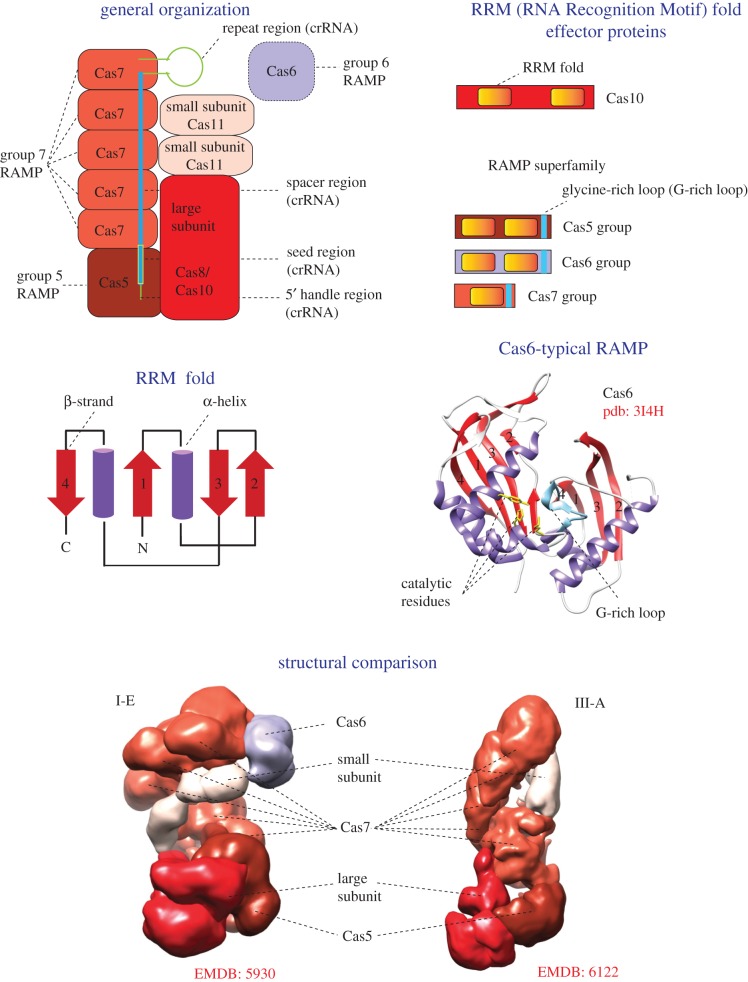
Key features and general organization of class 1 CRISPR-Cas systems. The figure schematically shows the general organization of a class 1 effector complex. The colouring of the shapes corresponds to the colour code for *cas* genes in figure 1. All proteins of class 1 effector complex that contain an RRM domain are schematically shown in a separate panel. The topology diagram of the RRM fold is schematically shown, with numbers corresponding to the typical order of β-strands in the fold. The Cas6 structure with two RRM fold domains, which are numbered according to the topology diagram, is shown as the ribbon diagram. The structural comparison of cryo-electron microscopy models demonstrates a striking similarity of effector complex organization of types I and III, despite the absence of significant sequence similarity between the corresponding subunits. The Electron Microscopy Data Bank (EMDB) codes are indicated for each structure. crRNA, CRISPR RNA; RAMP, Repeat-Associated Mysterious Protein. (Online version in colour.)

Despite limited direct sequence similarity, there is little if any doubt that the effector complexes of type I and type III that jointly compose the vast majority of class 1 systems share a common ancestry (figure 2). These complexes have strikingly similar overall architectures, and in both cases, the skeleton of the complex is formed by multiple copies of Cas7 protein, a member of the so-called RAMP (Repeat-Associated Mysterious Protein) superfamily [4,46,93,94]. Additionally, the effector complexes of both types contain a single copy of Cas5 protein, another RAMP superfamily member distantly related to Cas7, and the so-called large and small subunits (figures 1 and 2). Because of the generally fast evolution of *cas* genes and the resulting low sequence conservation [11], homology of the large and small subunits between types I and III could not be ascertained at the sequence level; however, the small subunits show significant structural similarity, which implies homology [95]. The large subunit of type III systems, Cas10, is a protein containing two RRM (RNA recognition motif) domains [96,97]. One RRM domain shows highly significant similarity to the palm domain, the catalytic domain of a broad variety of RNA and DNA polymerases and nucleotide cyclases [98,99]. This palm domain is predicted to be an active enzyme, whereas the second RRM domain is inactivated. The type I large subunit is the Cas8 protein, which is highly diverged in sequence even among different type I subtypes and shows no detectable sequence similarity, and only tenuous structural similarity, at best, to Cas10 [23]. This uncertainty notwithstanding, the conservation of the overall structural organization and the Cas7–Cas5 scaffold provides sufficient evidence for the common ancestry of the effector complexes between type I and type III (figure 2). Furthermore, considering that type I and type III systems together represent about 90% of all detected CRISPR-Cas loci, and moreover, amount to 100% among archaea [13], it is not much of a stretch to propose that the common ancestor of the type I and type III effector modules is also the ancestral form of this module for CRISPR-Cas systems in general [23,53]. By extrapolation, the ancestral effector module can be inferred to have contained, at least, the RAMP-based scaffold shared by the modern ones.

**Figure 3.**
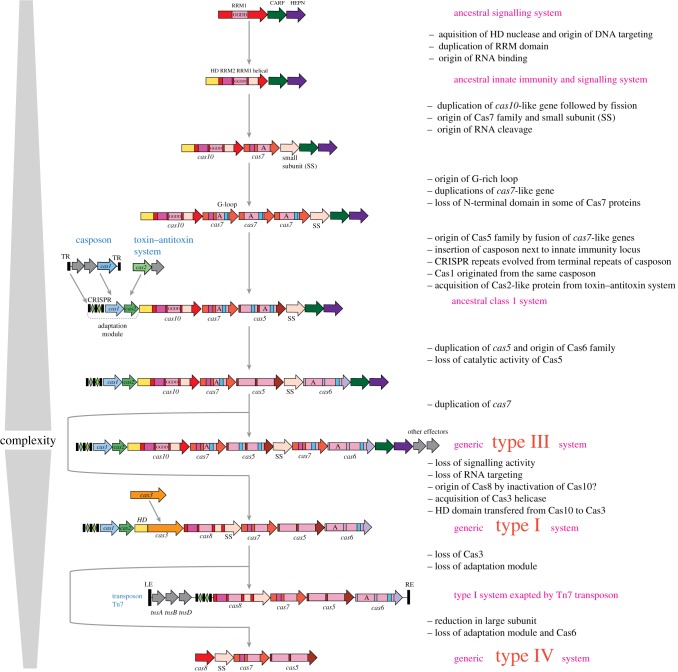
Origin and evolution of class 1 CRISPR-Cas systems. The figure depicts a hypothetical scenario of the origin of class1 CRISPR-Cas from an ancestral signalling system and its subsequent evolution yielding the extant type III and type I systems, as well as reductive evolution that produced type IV systems and minimalist variants of type I system recruited by Tn7 transposons. The key evolutionary events are described to the right of the images. ‘GGDD’, a key catalytic motif of the cyclase/polymerase domain of Cas10; ‘A’, catalytically active RRM domain of a RAMP protein; RE, LE, right and left end, respectively; TR, terminal repeats. (Online version in colour.)

Can we reconstruct the evolution of the effector module beyond the common ancestor of type I and type III? A key clue seems to be that, in type III, the entire effector complex, with the sole exception of the small subunit, is composed of domains with the same structural fold, the RRM fold, which is topologically identical to the widespread ferredoxin-like fold [19]. It seems likely, therefore, that the complex originally evolved by serial duplications and fusions of the RRM domains followed by subsequent extensive divergence (figures 2 and 3) [23]. The direction of evolution appears clear: from the RRM endowed with the polymerase–cyclase activity, as in Cas10, to the common ancestor of the RAMP superfamily which acquired a shared structural feature identifiable in all three groups of RAMPs, the glycine-rich loop (figures 2 and 3). Furthermore, structural comparisons suggest that small subunits from both type I and type III systems are homologous and similar to the C-terminal four-helix bundle domain of Cas10 [95,100]. Thus, it seems plausible that, following a duplication, fission of an ancestral Cas10-like protein could have given rise to the ancestors of both RAMPs and the small subunits (figure 3). Considering that catalytically active RAMPs of all three major families, Cas5, Cas7 and Cas6, are known, an ancestral RAMP also could have been an RNase (figure 3). However, independent origins of RNAse activity cannot be ruled out, especially considering that different Cas6 protein have different residues involved in catalysis (figures 2 and 3) [23,101–103].

Recent discoveries suggest clues also to the hardest puzzle, the organization and possible functions of the putative stand-alone ancestor of the CRISPR-Cas effector module. Most of the type III CRISPR-Cas systems encode proteins containing one or both characteristic domains: CARF (CRISPR-Associated Rossmann Fold), a (predicted) nucleotide-binding domain [104], and HEPN (Higher Eukaryote and Prokaryote Nucleotide-binding domain), an RNase that is primarily involved in various defence functions in both prokaryotes and eukaryotes [105]. The widespread examples of CARF–HEPN fusion in type III systems include Csm6 and Csx1; some type III systems instead include proteins that consist of a CARF domain fused to an unrelated nuclease or to a DNA-binding, HTH domain [104]. Years after the enzymatic activities of Cas10 and the HEPN domains as well as the nucleotide-binding capacity of the CARF domains, which implies allosteric regulation of the activity of HEPN (or other nucleases), have been predicted by computational methods [98,104,105], their functions in CRISPR-Cas have remain obscure, although it has been speculated that these proteins are linked through oligonucleotides synthesized by Cas10 [106].

One of the most notable findings in the CRISPR field in the last few years has been the experimental validation of this hypothesis. Two laboratories have independently demonstrated that a dedicated signalling pathway activated by target recognition is central to the immune function of type III CRISPR-Cas systems. Specifically, target binding by the crRNA–effector complex allosterically activates the polymerase activity of Cas10 which catalyses the synthesis of cyclic oligoA (cOA). The produced cOA molecules are bound by the CARF domain of the Csm6 protein (and by inference, other CARF domain-containing proteins), resulting in allosteric activation of the promiscuous RNase activity of the HEPN RNase domain of Csm6 and degradation of both the target RNA and other RNA molecules [107,108]. The outcome of this indiscriminate RNA degradation is thought to be the induction of dormancy or programmed cell death (PCD) in response to infection, which could work as a ‘contingency plan’ when immune response fails. Recently, it has been shown that this cOA–Cas10 signalling pathway is tightly regulated by cOA hydrolysis which is catalysed by CARF domains of a distinct family [109,110]. This regulatory circuit might be part of the microbial cell's sensor that ‘makes the choice’ between immunity and PCD [111,112].

The discovery of the cOA–Cas10 signalling pathway suggests a plausible possibility which, in fact, has been discussed previously solely on the basis of protein domain and gene neighbourhood analysis, that the ancestor of the CRISPR-Cas effectors was a stress-response system that triggered PCD upon activation by an alarmone, such as cOA [106,111] (figure 3). A potential candidate for the role of such a signal transduction system with a defence function has been identified in the genomes of several bacteria, namely, a protein that contains a single palm domain homologous to the active polymerase–cyclase domain of Cas10 [106]. The potential effector coupled to this predicted signalling enzyme remains unknown, although, in a few cases, both CARF and HEPN domains are fused to the minimal *cas10*-like gene [106]. Thus, the tight link between the three domains apparently emerged very early during evolution. Under the proposed model, the ancestral signalling system evolved to become an innate immunity mechanism as a result of the acquisition of an HD DNase domain and serial duplications, fusions and diversification of the RRM domains which yielded a complex endowed with the RNA-binding and -processing capacity (figure 3). The complexity of the organization of many type III loci encoding an active cOA synthetase and the growing number of ancillary proteins that are likely to be involved in the cOA signalling pathway as effectors imply a key role of the cOA pathway in defence mechanisms. Notably, in this model, the function of innate, and eventually, adaptive immunity evolved from a primordial PCD/dormancy induction form of defence (figure 3). These type III systems with dual functions are the most complex among all CRISPR-Cas systems. The loss of the signalling capacity through inactivation of the palm domain of Cas10 results in the loss of ancillary proteins, specialization on DNA targeting and the overall reduced complexity of the effector complex organization. This is the proposed course of evolution for type I systems as well as some type III variants (figure 3). Further reduction and loss of the DNA cleavage capacity, most probably, gave rise to the type IV CRISPR-Cas systems and other derivative CRISPR-Cas variants that were recruited by mobile elements to assist their replication and/or transposition (figure 3 and see below).

## 5. Class 2 effectors: multiple cases of nuclease recruitment from mobile genetic elements

**Figure 4.**
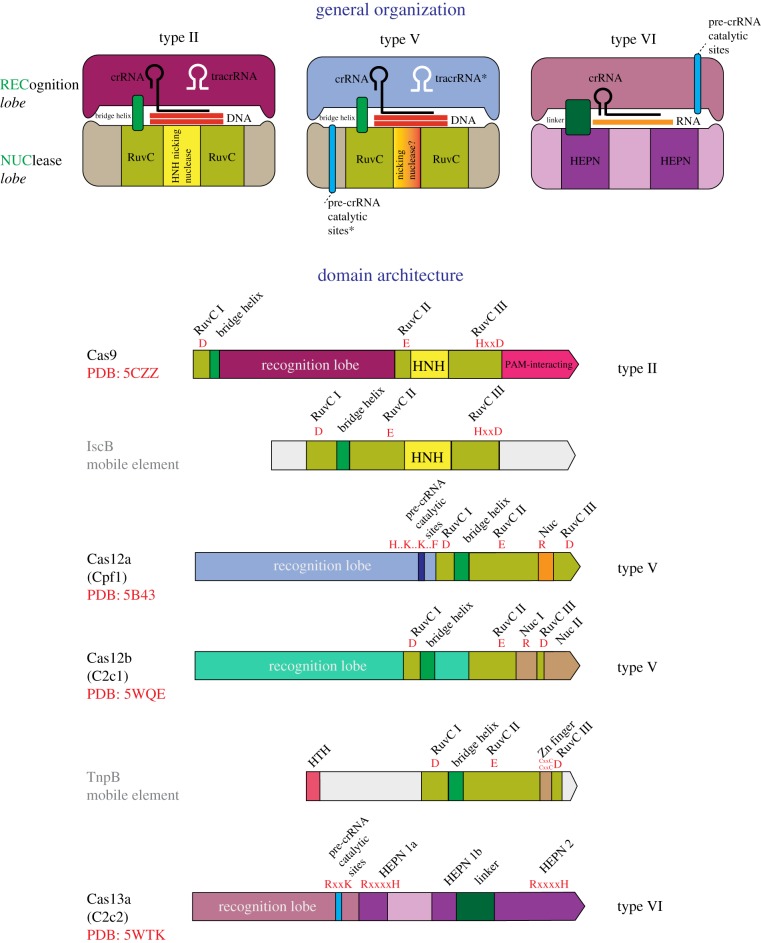
Key features, general organization and domain architectures of class 2 systems. Schematic of the complexes of effector proteins, with the target DNA or RNA, guide RNA and (for type II) tracrRNA shown on the top of the figure, and the domain architectures of the effector proteins depicted underneath. The catalytic residues of the effector nuclease domain and, for Cas12a and Cas13a, the residues shown to be required for pre-crRNA processing are indicated in red. The Protein Data Bank (PDB) codes are included for proteins with solved structures. HTH, helix–turn–helix DNA-binding domain. The tracrRNA, the pre-crRNA processing catalytic sites and the nicking, target strand-cleaving nuclease of the type V effectors are denoted by asterisks to indicate that they are each present only in subsets of the type II and type V effectors. The catalytic amino acid residues of the target-cleaving and pre-crRNA processing nucleases are shown in red. The small blue boxes show the approximate location of pre-crRNA processing nuclease domain. I, II, III are the distinct amino acid motifs that jointly compose the catalytic site of the RuvC-like nuclease. In the motif signature, ‘x’ stands for any amino acid, and ‘..’ indicates that the catalytic residues are separated by a small, variable number of non-conserved residues. Adapted from [8], with permission. (Online version in colour.)

Class 2 effectors are radically different from those of class 1 in that all the effector functions are concentrated in a single protein [13]. In-depth analysis of the protein sequences of class 2 effectors revealed a striking feature: they are all homologous to nucleases encoded by different classes of MGE [42,43]. All type II and type V effectors (Cas9 and Cas12 proteins, respectively) share a domain that belongs to the RuvC-like endonuclease family which belongs to the RNase H fold which is common to a great variety of nucleases and some other proteins [113] (figure 4). However, the sequence similarity between the RuvC-like domains of Cas9 and Cas12, and even between different subtypes within each type is low such that these proteins can be recognized as homologues only by highly sensitive sequence profile searches or structural comparisons [43,53]. Outside of the RuvC-like domain, the sequences of Cas9 and Cas12 show no similarity to each other and appear not to be homologous [43,53]. The structures of several Cas9 proteins [51,114–116], Cas12a (Cpf1) [66,67] and Cas12b (C2c1) [54,66] complexed with the guide RNA, target DNA and, in the cases of Cas9 and Cas12b, tracrRNA have been reported. All these effector proteins share similar size and overall shape which is a bilobed, ‘jaw-like’ structure accommodating the target DNA and the gRNA between the lobes. However, beyond the RuvC-like domains, the structures cannot be superimposed [117]. The RuvC-like domains of Cas9, Cas12a and Cas12b contain inserts, in similar but not identical positions, that represent non-homologous domains, respectively, the HNH family nuclease domain in Cas9 and unique, non-enzymatic domains that facilitate target cleavage in the Cas12 proteins [54,66,67,118].

An essential clue to the evolutionary origin of Cas9 and Cas12 was the observation that, apart from the members of the same family of Cas protein, both show the highest sequence similarity to the RuvC-domain-containing TnpB proteins of the IS*605* and other related families of transposons [42,53]. The *tnpB* genes are among the most abundant genes in bacterial and archaeal genomes, and are encoded either by autonomous transposons, which additionally encode a transposase (TnpA), or more frequently, by non-autonomous transposons, including the eukaryotic Fanzor elements, in which TnpB is the only protein product [119]. The role of TnpB in transposons remains unclear, given that this protein is not required for transposition and, actually, appears to downregulate it [120], but the perfect conservation of the RuvC-like endonuclease catalytic sites in most TnpB sequences indicates that these proteins are active nucleases.

**Figure 5.**
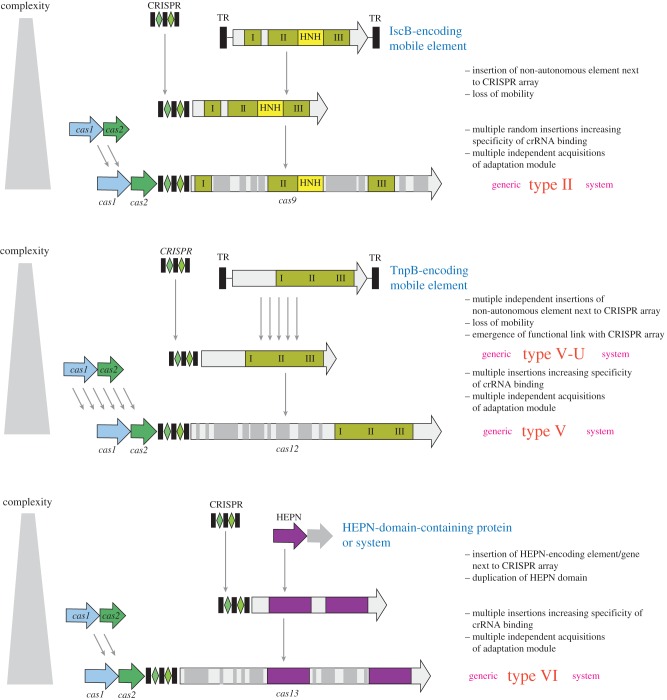
Origin of the class 2 CRISPR-Cas effectors from MGE. The figure depicts a hypothetical scenario of the origin of class 2 CRISPR-Cas from non-autonomous transposons, for type II and type V systems, and from a defence system (toxin–antitoxin module) for type VI systems. IscB and TnpB are the inferred ancestors of the type II (Cas9) and type V (Cas12) effectors, respectively. Inserts that could have contributed to increased specificity and efficiency of the effectors are shown by grey rectangles of variable size. I, II and III are the distinct amino acid motifs that jointly compose the catalytic site of the RuvC-like nuclease. TR, terminal repeats. (Online version in colour.)

Unexpectedly, the effectors of type II and different subtypes of type V (Cas9 and Cas12a, 12b, 12c, respectively) showed the highest similarity to different groups of TnpB proteins, suggesting independent origins from the same ancestral protein family [53]. Because of the low sequence conservation, no reliable phylogenetic trees could be constructed for the type II and type V effectors together with the TnpB proteins. However, the ancestry of Cas9 could be readily traced to a distinct family of transposons (denoted ISC, after Insertion Sequences Cas9-related) that are found primarily in Cyanobacteria and encode IscB proteins which share a signature domain architecture with Cas9, namely, the insertion of an HNH endonuclease domain into the TnpB (RuvC-like) domain [42,121] (figure 5).

The likely path of evolution from TnpB to the type V effectors became more tractable with the identification of a distinct variety of putative CRISPR-Cas systems that were designated subtype V-U (after uncharacterized). Subtype V-U loci lack adaptation modules and typically consist solely of TnpB homologues encoded next to CRISPR arrays [43]. The putative V-U effectors are much smaller than Cas9 or Cas12 but are similar in size or only slightly larger (400–600 amino acid residues) than typical, transposon-encoded TnpB proteins. In contrast with Cas9 and Cas12, the TnpB homologues encoded in the V-U loci are strongly similar to the transposon-encoded TnpB. Accordingly, the V-U proteins could be included in robust phylogenetic trees which convincingly support independent origins of (at least) five distinct groups of putative V-U2 effectors from different TnpB subfamilies [43]. The experimental demonstration of the functionality of the type V-U systems is pending. However, the evolutionary conservation of five distinct variants of subtype V-U in diverse bacteria, the finding that the spacer sequences are completely different even in closely related V-U loci and the presence of multiple phage-specific spacers jointly show that at least some V-U variants are functional CRISPR-Cas systems [43].

The close similarity between the predicted V-U effectors and transposon-encoded TnpB proteins implies that subtype V-U represents recently evolved, ‘baby’ CRISPR-Cas systems. The evolutionary scenario for type II and type V systems (figure 5) starts with random insertion of the non-autonomous TnpB-encoding transposons next to CRISPR arrays. Given the enormous abundance of these transposons in bacterial and archaeal genomes, such random insertions can be expected to occur frequently, and indeed, apart from the evolutionarily conserved V-U variants, a number of apparently spurious juxtapositions of CRISPR arrays and *tnpB* genes have been detected [43]. The subsequent evolution would involve parallel, independent ‘maturing’ of the effectors via acquisition of additional domains the sources of which remain obscure and might have involved both internal duplications and recombination [43] (figure 5). The gained portions of the proteins are unrelated between different subtypes, but the convergent outcome is the emergence of an effector protein that is large and flexible enough to accommodate the complex of the crRNA with the target DNA (figure 5). It should be emphasized that not only the effector modules but also the adaptation modules of class 2 systems were acquired independently from different class 1 variants (figure 5) [43].

In addition to two distinct classes of MGE, casposons and TnpB-encoding transposons, microbial toxin–antitoxin (TA) modules seem to have been important contributors to the evolution of CRISPR-Cas systems. The most common type II TA modules consist of two proteins, a toxin and an antitoxin, of which the antitoxin is substantially less stable than the toxin and is eliminated by proteolysis under stress, resulting in toxin activation [122–126]. The most common toxin domains are the interferases, RNases that indiscriminately cleave mRNAs inside the ribosome, resulting in microbial dormancy or cell death [127,128]. The interferases belong to several unrelated protein families including HEPN, RelE and VapD, a distinct variant of the RRM fold [105,124]. Although the TA modules lack mechanisms of active mobility, they nevertheless qualify as MGE because they are typically transferred on plasmids and are ‘addictive’ to the host cells, which die if they do not receive the TA-carrying plasmid upon segregation owing to the difference in stability between the toxin and antitoxin proteins [123,125,126]. In addition to plasmids, many TA loci are found in bacterial and archaeal chromosomes, and are thought to induce dormancy or PCD as an ‘altruistic’ defence strategy [129,130].

**Figure 6.**
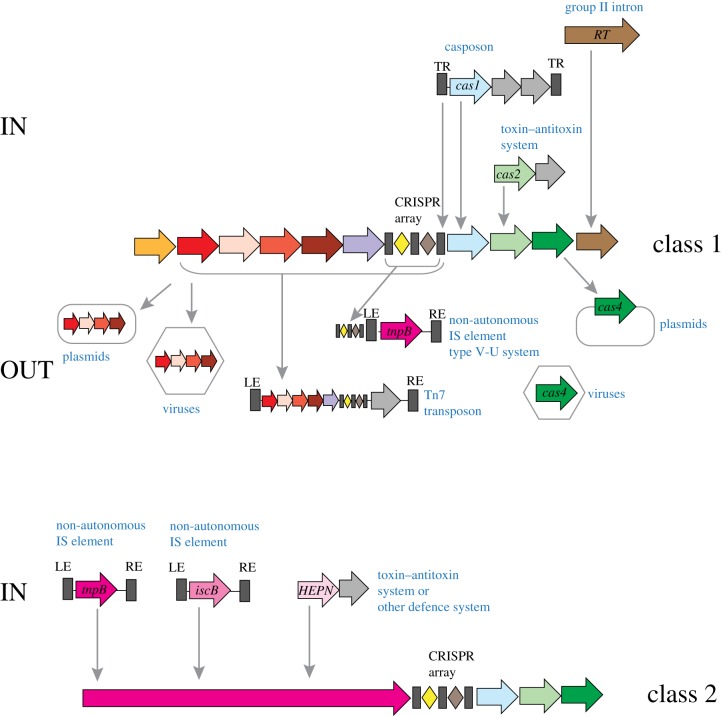
‘IN and OUT’: exchange of components between CRISPR-Cas systems and MGE. The figure shows a hypothetical scenario of *cas* gene acquisition by evolving CRISPR-Cas systems from MGE (IN) and by MGE from CRISPR-Cas systems (OUT). Genes are shown by arrows. The colouring corresponds to distinct *cas* genes and is the same as in figure 1. Grey arrows denote any genes that are considered to be unrelated to CRISPR-Cas. Specific acquisition events are shown for class 1 and class 2 systems separately. Arrows indicate the inferred direction of the gene flow. HEPN, RNase of the HEPN (Higher Eukaryotes and Prokaryotes Nucleotide-binding domain) superfamily; RE, LE, right and left end, respectively; RT, reverse transcriptase; TR, terminal repeats. (Online version in colour.)

At least two unrelated classes of TA appear to have contributed to the evolution of CRISPR-Cas. The structural subunit of the adaptation complex, Cas2, belongs to the VapD family of interferases [19]. The interferase catalytic site is intact in the majority of the Cas2 proteins but is disrupted in some and is not required for adaptation [36]. Thus, the role of the demonstrated nuclease activity of Cas2 [131–133] in CRISPR-Cas function remains uncertain, and it cannot be ruled out that Cas2 functions as a toxin inducing dormancy or PCD when the immune function of CRISPR-Cas fails [112,134]. As discussed above, the *cas2* gene might have become a component of CRISPR-Cas systems via a casposon that gave rise to the adaptation module (figures 3 and 6). Given that some casposons encode also nucleases homologous to Cas4 [83,90], this Cas protein that is part of the adaptation module in several CRISPR-Cas subtypes also could originate from that ancestral casposon.

As also discussed above, the HEPN domain, the other abundant toxin RNase, is present in many Cas proteins in type III and in all type VI systems. In type III systems, the HEPN domains present in accessory proteins, such as Csm6 and Csx1, are the endpoint RNA-cleaving effectors of the cOA–Cas10 signalling pathway and likely originate from the ancestral signal transduction module (see above; figure 3). The type VI effectors (Cas13) are large proteins containing two diverged HEPN domains that are both required for target RNA cleavage and the promiscuous RNase activity that is activated by target binding [43,53,59,68,69]. The presence of two HEPN domains is a unique signature of the type VI effectors that testifies to their common origin despite the extreme divergence of the HEPN domain sequences and the lack of detectable sequence similarity outside of these domains. The extremely low sequence conservation typical of the HEPN domains precludes reliable phylogenetic analysis and thus confident identification of the specific ancestors. Thus, it is unclear whether the HEPN domains in type VI effectors originate from type III HEPN-containing proteins or, independently, from toxins. Regardless, a ‘maturing’ path of evolution resembling that proposed for the type II and type V effectors appears likely for Cas13 (figure 5).

## 6. Origin of reverse transcriptase-containing adaptation modules for group II introns

A large subset of type III CRISPR-Cas systems, in addition to the regular process of spacer capture from foreign DNA, are capable of acquiring spacers from RNA (typically, transcripts of an invading DNA genome) that is reverse-transcribed by a CRISPR-associated RT [29]. As pointed out above, the RT is a component of a distinct variety of adaptation modules that have been shown to combine promiscuously with diverse variants of type III effector modules or found in a stand-alone form, adjacent to a CRISPR array [30]. In these adaptation modules, the RT is often fused to Cas1 or, alternatively, is encoded by a gene adjacent to *cas1*. Phylogenetic analysis of the RT superfamily shows that most of the CRISPR-associated RTs form a strongly supported clade that is affiliated with the RTs of group II introns [30,135,136]. Thus, the RT–Cas1 fusion represented in diverse type III loci appears to have emerged at a single point in evolution, conceivably, as a result of a random insertion of a group II intron into a type III CRISPR-*cas* locus (figure 6). There is a clear, striking parallel between this evolutionary scenario and that for the origin of type II and V effectors from TnpB-encoding transposons.

## 7. Derived CRISPR-Cas variants: reductive evolution and exaptation for non-defence functions

In addition to the diversification of the immune functions, an emerging trend in the evolution of CRISPR-Cas systems is the emergence of derived, defective variants that lose the adaptation and interference capacities, and are exapted for roles other than adaptive immunity. In particular, such minimalist CRISPR-Cas variants are carried by transposons and plasmids [137] (figures 3 and 6). The most common of these is a minimal version of subtype I-F that is encoded by a large family of Tn7-like transposons; smaller groups of Tn7-like transposons encode similarly degraded subtype I-B systems [138]. Phylogenetic analyses of both Tn7 genes and Cas7, the most highly conserved protein in these minimalist CRISPR-Cas systems, identified a single event of subtype I-F capture by a transposon and two independent events of subtype I-B acquisition [138]. All these Tn7-encoded CRISPR-Cas variants lack both the adaptation module and the Cas3 protein that is required for target cleavage by type I systems and, accordingly, are incapable of either adaptation or interference. However, they encompass all the subunits of the pre-crRNA processing complex and therefore can be inferred to generate mature crRNAs and recognize the target DNA (figure 3). The Tn7-encoded CRISPR-Cas loci encompass short CRISPR arrays, some of which contain spacers that target plasmids, bacteriophages sharing hosts with the respective transposons or chromosomal sequences adjacent to integration sites. The transposon-encoded CRISPR-Cas systems remain to be studied experimentally. Nevertheless, the predicted ability of these systems to recognize but not to cleave cognate targets suggests the intriguing possibility that they facilitate insertion of transposons into MGE by generating R-loops at the target sites [138]. In evolutionary terms, the transposon-encoded CRISPR-Cas systems clearly are derived forms that evolved from the respective complete systems.

Type IV CRISPR-Cas systems represent another minimalist variant unrelated to those carried by Tn7-like transposons. Type IV loci are typically carried by plasmids and, in some cases, by prophages of diverse bacteria. Analogous to the transposon-encoded type I variant, type IV systems, with a few exceptions, lack an adaptation module and consist of *cas5*, *cas7* and *cas8* genes, and in certain cases, also cas6, along with an additional gene, which in different type IV variants encodes either a DinG family DNA helicase or an uncharacterized small protein [8,13]. Type IV loci rarely include CRISPR arrays and accordingly can be predicted to use *in trans* arrays located in another region of the same plasmid away from or on the host chromosome. As with the transposon-encoded variants, the functions of type IV CRISPR-Cas systems remain obscure but, given their almost exclusive localization on plasmids, it should be expected that they facilitate maintenance and/or enhance the mobility of plasmids via as yet unknown mechanisms.

A recent systematic screening of microbial genomes for CRISPR-linked genes [139] has led to the discovery of an apparently defective variant of I-E systems that lacks the *cas3* gene and, by implication, cannot cleave targets. Instead, these loci encode an NTPase of the STAND (Signal Transduction ATPases with Numerous Domains) superfamily [140] which is implicated in signalling processes, possibly stress-induced PCD.

The subtype V-U loci discussed above also constitute a variety of ‘minimal’ CRISPR-Cas systems. One of the five distinct groups within subtype V-U, V-U5, encompasses a TnpB homologue that is predicted to be inactivated as a result of the replacement of the catalytic amino acid residues in the RuvC-like nuclease domain [43]. Thus, this system is predicted to perform functions that do not involve target cleavage.

Taken together, these examples show that the reductive evolution of CRISPR-Cas systems leading to their exaptation (recruitment) for non-defence (or at least, not involving target cleavage) functions occurred on multiple independent occasions. So far, none of these functions has been explored experimentally, so the study of these systems appears to be a wide-open research direction that is bound to yield new insights into microbial physiology. More instances of defective CRISPR-Cas systems can be expected to emerge with the advances of genomic and metagenomics, particularly, in MGE genomes.

## 8. The emerging synthesis on CRISPR-Cas evolution

The CRISPR-Cas systems are highly complex molecular ensembles, and as such, undoubtedly are products of a complicated succession of evolutionary events. Moreover, as with all complex systems, the spectre of irreducible complexity looms: a satisfactory evolutionary scenario is expected to account for the functionality of intermediate stages [141–144]. Perhaps, surprisingly, we believe that the findings discussed above provide enough clues for a plausible overall scenario (figures 3, 5 and 6).

The single over-arching theme of CRISPR-Cas evolution is the evolutionary entanglement between these systems of microbial adaptive immunity and various types of MGE. Strikingly, at least four unrelated MGE varieties have contributed to CRISPR-Cas evolution: (i) casposons that gave rise to the adaptation module, (ii) group II introns that donated the RT to a distinct variety of type III adaptation modules, (iii) non-autonomous IS*605*-like transposons, the ancestors of type II and type V effectors, and (iv) a TA module that apparently contributed Cas2.

The evolution of class 2 CRISPR-Cas systems clearly involved multiple acquisitions of ancestral MGE genes encoding nucleases that subsequently evolved into CRISPR-Cas effectors. In all likelihood, these genes were captured as a result of chance insertion of the respective MGE into pre-existing CRISPR-*cas* loci or next to orphan CRISPR arrays [43,137]. A puzzling aspect of this part of CRISPR-Cas evolution is the switch of the pre-crRNA processing from the Cas6-mediated mechanism characteristic of class 1 to the effector-catalysed and tracrRNA-dependent mechanisms in class 2. The tracrRNA which is required to recruit RNase III for processing apparently evolved on multiple occasions in different type II and type V systems [145], suggesting that the autonomous, effector-dependent processing is ancestral in class 2. The provenance of this mechanism remains an enigma on which the detailed study of the ancestral MGE-encoded nucleases might shed light.

Importantly, the evolutionary link between CRISPR-Cas and MGE is a two-way street: complete CRISPR-Cas loci, their reduced versions or individual components were repeatedly recruited by various MGE and adopted for antidefence as well as, apparently, for other, still uncharacterized functions.

When the evolution of class 1 effector modules is considered, the second key theme comes up, namely, serial duplication of ancestral genes followed by extreme diversification (figure 3). The ancestral unit is the RRM domain. The expansive RAMP superfamily undoubtedly evolved via a series of RRM duplications. We postulated that the founder of this superfamily also emerged through a duplication of the ancestral, enzymatic RRM domain in the evolutionary progenitor of Cas10, but this connection remains tenuous. The core of the ancestral effector complex could have been a signals transduction system consisting of the ancestor of Cas10 (a cOA polymerase) and a CARF–HEPN effector that triggered dormancy or PCD in response to infection or other forms of stress. This part of the CRISPR-Cas evolution scenario is, admittedly, the weakest and requires the most effort in genome mining and structural comparison to complete the reconstruction convincingly.

The third major trend is the reductive evolution of CRISPR-Cas systems which led to defective variants that apparently were recruited for functions other than adaptive immunity. Notably, many if not most of such variants reside in MGE [137] and, presumably, contribute to the reproduction of those elements, although the mechanisms involved remain enigmatic.

On a more general plane, the contributions of MGE to the evolution of an adaptive immunity system and, conversely, the recruitment of defence systems or their components by MGE for antidefence (as in some bacterial viruses that encode complete CRISPR-Cas systems) or other functions (as in the case of defective systems discussed here) fits the ‘guns for hire’ concept [146]. Within this framework, enzymes are shuttled between defence systems and MGE, going to the ‘highest bidder’ (the elements providing the highest reproductive benefit to the respective genes), given that the activities involved, such as those of transposase, site-specific or promiscuous (in the case of TA) nuclease, helicase or RT, are closely analogous if not identical. This concept is applicable far beyond CRISPR-Cas: for example, different, unrelated transposons were the ancestors of key components of the adaptive immunity system in vertebrates [41] and DNA diminution system in ciliates [147].

## Data accessibility

This article has no additional data.
